# A Model Analysis of Mechanisms for Radial Microtubular Patterns at Root Hair Initiation Sites

**DOI:** 10.3389/fpls.2016.01560

**Published:** 2016-10-28

**Authors:** Pawel Krupinski, Behruz Bozorg, André Larsson, Stefano Pietra, Markus Grebe, Henrik Jönsson

**Affiliations:** ^1^Computational Biology and Biological Physics, Department of Astronomy and Theoretical Physics, Lund UniversityLund, Sweden; ^2^Department of Plant Physiology, Umeå Plant Science Centre, Umeå UniversityUmeå, Sweden; ^3^Institute of Biochemistry and Biology, Plant Physiology, University of PotsdamPotsdam, Germany; ^4^Sainsbury Laboratory, University of CambridgeCambridge, UK; ^5^Department of Applied Mathematics and Theoretical Physics, University of CambridgeCambridge, UK

**Keywords:** plant cell wall, finite element modeling, computational morphodynamics, root hair initiation, microtubules, cellulose fibers, composite material

## Abstract

Plant cells have two main modes of growth generating anisotropic structures. Diffuse growth where whole cell walls extend in specific directions, guided by anisotropically positioned cellulose fibers, and tip growth, with inhomogeneous addition of new cell wall material at the tip of the structure. Cells are known to regulate these processes via molecular signals and the cytoskeleton. Mechanical stress has been proposed to provide an input to the positioning of the cellulose fibers via cortical microtubules in diffuse growth. In particular, a stress feedback model predicts a circumferential pattern of fibers surrounding apical tissues and growing primordia, guided by the anisotropic curvature in such tissues. In contrast, during the initiation of tip growing root hairs, a star-like radial pattern has recently been observed. Here, we use detailed finite element models to analyze how a change in mechanical properties at the root hair initiation site can lead to star-like stress patterns in order to understand whether a stress-based feedback model can also explain the microtubule patterns seen during root hair initiation. We show that two independent mechanisms, individually or combined, can be sufficient to generate radial patterns. In the first, new material is added locally at the position of the root hair. In the second, increased tension in the initiation area provides a mechanism. Finally, we describe how a molecular model of Rho-of-plant (ROP) GTPases activation driven by auxin can position a patch of activated ROP protein basally along a 2D root epidermal cell plasma membrane, paving the way for models where mechanical and molecular mechanisms cooperate in the initial placement and outgrowth of root hairs.

## 1. Introduction

Most higher plants do not display cell migration and need to generate optimal shapes by adjusting growth both in terms of magnitude and directions. Two main modes of growth are prevailing across the plant kingdom (Baskin, 2005; Rounds and Bezanilla, 2013). The first is diffuse growth where whole cells or tissues are expanding quite homogeneously, although often anisotropically. The other mode of growth is tip growth, where expansion appears in a focused region of a cell. The growth is dependent on environmental signals and guided by cells, genetic and hormonal interactions (Chen et al., 2016). Still, to effectuate the growth, manipulation of the stiff cell walls surrounding all cells is necessary (Cosgrove, 2005).

The plant cell wall can be seen as a complex composite material composed mainly of cellulose microfibrils, pectins and xyloglucans (Baskin, 2005; Cosgrove, 2005). Intricate connections between these wall components and their effect on the mechanical properties of the cell wall are not yet completely understood. Similarly, the way in which the plant dynamically controls composition and properties of its cell walls to form different organs to their appropriate shape is a matter of extensive research (Braybrook and Jönsson, 2016). Cortical microtubules serve as the guiding tracks for deposition of cellulose microfibrils and in consequence cells can control anisotropy of their wall stiffness (Arioli et al., 1998; McFarlane et al., 2014). This in turn relates to directionality of anisotropic growth of a tissue and influences stresses at subcellular to tissue scales (Green, 1962; Heath and Geitmann, 2000; Baskin, 2005). For tip-growing root hairs, the cellulose fibers have been shown to be randomly oriented at the very tip, while organized longitudinally away from the tip where there is also a formation of a secondary wall (Newcomb and Bonnet, 1965; Park et al., 2011; Akkerman et al., 2012). In tip growth, high rates of wall material deposition are promoting the localized growth (Geitmann et al., 2000).

Several signals regulating the dynamic orientations of the cortical microtubules have been suggested, including environmental, molecular and mechanical regulation (Hogetsu, 1986; Zandomeni and Schopfer, 1993; Hamant et al., 2008; Lindeboom et al., 2013; Chen et al., 2014, 2016), and for diverse input signals microtubule severing is an important part of the orientation process as shown by katanin mutants (Uyttewaal et al., 2012; Lindeboom et al., 2013; Chen et al., 2014; Sampathkumar et al., 2014; Sassi et al., 2014). The *Arabidopsis* hypocotyl displays a strong growth response to light. Hypocotyl microtubules were recently shown to quickly reorient from transverse to longitudinal after being exposed to blue light and this reorganization was dependent on katanin (Lindeboom et al., 2013). Treatment with the phytohormone auxin has been shown to induce changes in microtubule orientations (Zandomeni and Schopfer, 1993), which more recently has also been reported for *Arabidopsis* roots and hypocotyls (Chen et al., 2014). Again the reorientation is quick (Chen et al., 2014), but it is yet to be understood whether growth is affected in such treatments (Baskin, 2015).

For several of the suggested input cues orienting microtubules it is unclear how the input provides a directional signal. Mechanical stresses and strains could serve that purpose. Mechanical stresses in the walls have been suggested to provide a directional signal where cortical microtubules orient along the maximal principal stress direction (Hejnowicz et al., 2000), both at the tissue and at the subcellular levels in shoots, leaves and flowers in *Arabidopsis* (Hamant et al., 2008; Sampathkumar et al., 2014; Hervieux et al., 2016). Such feedback loop between stress and direction of material anisotropy has been implemented in models which have verified its ability to produce robust regulation of anisotropic growth (Bozorg et al., 2014). In particular, such a model correctly predicts the circumferential arrangement of microtubules (and tissue scale stresses) around the sites of primordia outgrowth in the shoot apical meristem and toward the stem tissue. The question of how plant cells can sense mechanical stress remains unanswered. In principle stresses can be measured through deformation of microscopic cell wall or membrane components, but the direct confirmation of such mechanism is still lacking.

In tip growing cells, the growth is much more localized to a specific site of the cell wall. As mentioned above, the microtubules are randomly organized at the tip, and growth is rather promoted by vigorous local deposition of the new material to the site of outgrowth. At the tip there is a region of the cytosol less abundant in large organelles and with targeted secretion of wall material seen by enriched presence of secretory vesicles (Galway et al., 1997; Lovy-Wheeler et al., 2007; Rounds and Bezanilla, 2013). Pectin deposited to the tip is further de-esterified and rigidified by calcium cross-linking, promoted by high levels of calcium at the tip (Sanati Nezhad et al., 2014). In particular, the addition of wall material, and hence the cell wall thickness at the tip, is oscillating and is out of phase with growth rates, alternating thick walls with high growth rates (McKenna et al., 2009). Also actin has been shown to play a prominent role in wall elongation processes (Geitmann et al., 2000). When measuring the rigidity of pollen tubes using cellular force microscopy, the apparent reduced stiffness at the tip was attributed to the respective geometrical change (Vogler et al., 2013). Computational models of tip growth connect deformation to the addition of material, the use of anisotropic wall material, and strain-based growth (Dumais et al., 2006). In addition, the inclusion of pectin chemistry provides means to have parameter space regions determining steady and oscillatory growth in such model (Rojas et al., 2011). Moreover, models including details of osmotic pressure alterations discuss possible roles of pressure as a driving force for oscillatory tip growth (Hill et al., 2012), as suggested by experimental data (Zonia, 2010), although controversial (Winship et al., 2010).

We are particularly interested in the process of root hair initiation. A transcriptional network for root hair cell differentiation in *Arabidopsis* has been identified (Schiefelbein et al., 2009), defining alternating cell files of root hair cells (trichoblasts) and non root hair cells (atrichoblasts). The differentiation of root hair cells has been modeled, suggesting different mechanisms (Savage et al., 2008; Benítez and Alvarez Buylla, 2010; Benítez et al., 2011). While root hair initiation often fails in genetic perturbations of components of these networks, these proteins are not known to provide information on the polar position of root hair initiation sites on the lateral membrane of epidermal cells. Similarly, auxin has been suggested to identify files of root hair cells. Its supply is facilitated, at least in part, by auxin influx mediators throughout non root hair cells files (Jones et al., 2009).

More interesting for the subcellular localization of the root hair is that an intracellular auxin gradient has been proposed to be informative in the positioning of root hairs on the lateral membrane of hair cells, close to their basal (rootward) end (Fischer et al., 2006). One of the earliest markers of the basal initiation site are the activated Rho-of-plants (ROP) GTPases (Molendijk et al., 2001; Jones et al., 2002; Xu and Scheres, 2005; Fischer et al., 2006). The ROP localization has also been found to correlate with positioning of lobes and necks in pavement cells where ROP is activated by auxin (Xu et al., 2010). The ROP proteins are likely to be important for the correct placement and outgrowth of root hairs as suggested by dominant-interference and overexpression studies (Molendijk et al., 2001; Jones et al., 2002). The activation dynamics of ROP proteins in root hair cells has been modeled using a reaction-diffusion type of model where auxin at the subcellular level is assumed to promote activation of ROP (Payne and Grierson, 2009), similar to models of Rho GTPases generating spontaneous intracellular patterns in other organisms (Jilkine et al., 2007; Goryachev and Pokhilko, 2008). In the former, reaction-diffusion model, ROP was explicitly divided into an active and an inactive form. Active ROP was further assumed to be bound to the membrane, while the inactive form was assumed to be located in the cytosol, implemented as a lower diffusion rate of the active form of ROP compared to the inactive form of ROP. Together with a positive self-feedback of ROP-activation this was sufficient to generate peaks of activated ROPs at the root-tip oriented (basal) end of cells in a 1D model, predicting the positioning of root hair initiation in wild type as well as in selected mutants. Detailed investigations of the ROP patterning model have revealed a possibility for more complex dynamics, where ROP peaks can move transiently, and the patterning dynamics of the model have been shown to exhibit hysteresis behavior (Brena-Medina et al., 2014).

**Figure 1:**
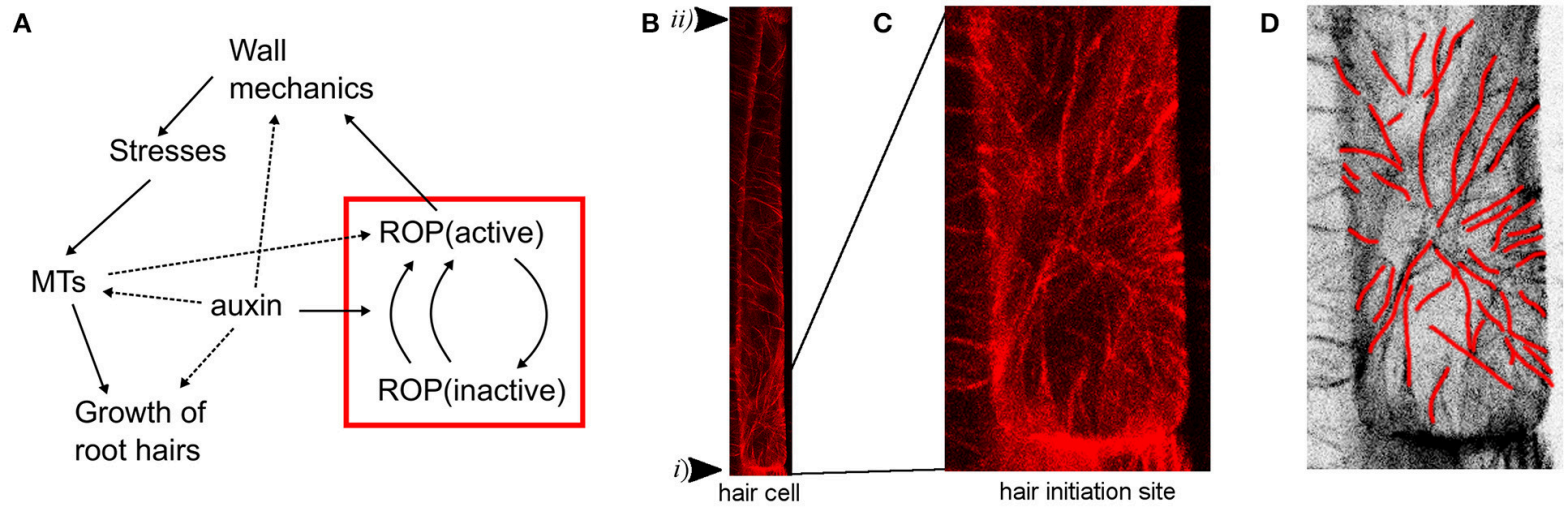
**Root hair initiation is dependent on ROP activation and microtubular dynamics**. **(A)** Model diagram of the root hair initiation process including feedback between cycling of ROPs and mechanical properties via microtubular dynamics. Solid lines represent mechanisms explicitly modeled, dashed lines are implicitly in the models or suggested in the literature. The red box represents the ROP model tested in 2D simulations and the arrow from the ROP to mechanics is evaluated by testing different hypotheses. **(B)** Pattern of cortical microtubules in a root epidermal cell visualized by RFP-TUB6, underlying the root epidermal cell membrane of a 5-day-old *Arabidopsis thaliana* seedling. The arrowheads mark basal (i) and apical (ii) ends of the cell. In the top part a mainly transverse pattern of microtubules can be seen, while a star-like radial pattern around the root hair initiation site can be seen at the lower end. **(C)** Magnification of the radial pattern in the lower part of the cell from **(B)** showing root hair outgrowth site. **(D)** Microtubules directions marked with red lines in inverted image of **C**.

Also the actin and microtubular cytoskeleton networks are important for correct root hair formation (Bao et al., 2001; Ringli et al., 2002; Kiefer et al., 2015). When microtubules were imaged together with PIP5K3, an early root hair initiation marker (Kusano et al., 2008), microtubules were reported to orient into a radial pattern surrounding the root hair initiation site (Pietra et al., 2013). Similar to other microtubule organizing events, this was disrupted in mutants defective in the *SABRE* and *CLASP* genes required for microtubule organization. Also, the basal positioning of root hairs as well as the polar localization of the ROP patches were perturbed in different combinations of loss-of-function mutants, indicating a regulatory role of microtubular patterning for polar ROP placement. Consistent with this view, the *procuste1*/*cesa6* mutant defective in a cellulose synthase subunit displays alterations in polar ROP and root hair placement (Singh et al., 2008), resembling the defects in *sabre* mutants and suggesting a requirement for both correct microtubule organization and cellulose microfibril synthesis during polar root hair initiation. In addition, ROPs have been reported to be activated by auxin and regulate microtubular patterning in pavement cells (Fu et al., 2009; Xu et al., 2010). Hence, an intricate feedback mechanism between ROPs and microtubules connecting also auxin and wall mechanics seems to be at the core of root hair initiation and growth (Figure 1A).

Altogether, the ROP and microtubular data indicate a complex feedback between molecular and cytoskeletal dynamics during root hair initiation, and computational modeling is essential to understand the behavior. In particular, current data raise the question if a correlation between microtubule organization and principal stress direction is sustained in the case of the root hair initiation, as has been observed before in diffuse growth (Figure 1A). In diffuse growth of organ formation, auxin is accumulated at the site of outgrowth, leading to the loosening of cell wall material. In effect, we observe around the outgrowth region circumferential stress orientation and corresponding microtubule pattern. Here, we extend the previously published 1D ROP model to 2D to confirm it can provide a mechanism for correct placing of an activated ROP patch along a root hair cell. We then investigate whether the previously suggested mechanical stress feedback on microtubule directions can predict the patterns seen at root hair initiation sites by analyzing mechanical scenarios of tip growth that can produce radial stress patterns.

## 2. Methods

### 2.1. Plant growth and imaging

Plant growth medium and conditions were as described (Fischer et al., 2006). Seeds were surface sterilized and stratified at 4°C for 3 days before plating on MS plates (1 × MS medium, 1% sucrose, 0.8% plant agar, 1 M morpholinoethanesulfonic acid, pH 5.7). Seedlings were grown vertically at 23°C day and 18°C night under 16 h light/8 h dark photoperiod and subjected to analysis after 5 days. Confocal imaging followed (Pietra et al., 2013). Cortical microtubules were imaged in epidermal cells of seedlings expressing pUBQ1:RFP-TUB6 (Ambrose et al., 2011). Z stacks of planes at 0.53 μ*m* distance intersecting the periclinal face of the cell were acquired and employed to generate maximum intensity projections.

### 2.2. ROP activation model

We developed a ROP activation model based on a previously published model (Payne and Grierson, 2009). The model describes the ROP dynamics in 2D close to the epidermal cell membrane of a root trichoblast. The ROP activation is influenced by an auxin (*A*) gradient, which in our case is produced by a source-sink model in which auxin is allowed to diffuse and is subject to a constant degradation rate. Based on the assumption of a basipetal auxin flow in the epidermis (due to reported fluxes and gradients), auxin is produced in the basal part of the cell, representing auxin influx, and degraded at the apical side of the cell, representing auxin outflux. The auxin dynamics are described by

(1) $$\begin{array}{rcl} \frac{dA}{dt} & = & D_{a}\Delta A+t+s_{in}-s_{out}A-qA \end{array}$$

where *s*_*in*_ is the auxin production at the source and *s*_*out*_ is the auxin degradation at the sink. Further, *q* is the auxin degradation rate, *D*_*a*_ is the rate of auxin diffusion and *t* is a general auxin production. For the simulation with an auxin gradient, the parameter *t* is set to zero (Table 1). In the simulations with constant auxin levels, all parameters are zero except for the general production rate *t* and the degradation rate *q*. The ROPs can be in an “inactive” form (*R*_*i*_) moving in the cytosol, or in an “activated” form (*R*_*a*_) where they sit in the membrane less prone to move. In addition to a constant activation of the ROPs with rate *k*_1_ and an inactivation with rate *c* there is also an auxin-dependent activation with the rate *k*_2_ that depends also on the active ROP concentration, creating a positive feedback. The full ROP dynamics are described by

(2) $$\frac{dR_{a}}{dt}=\{\begin{array}{l} D_{1}\Delta R_{a}+a+R_{i}\cdot \left(k_{1}+k_{2}R_{a}^{2}A\right)-cR_{a}-(r+p)R_{a}\\ \text{ if boundary​}\\ D_{1}\Delta R_{a}+a+R_{i}\cdot \left(k_{1}+k_{2}R_{a}^{2}A\right)-cR_{a}-rR_{a}\\ \text{ otherwise} \end{array}$$

(3) $$\begin{array}{rcl} \frac{dR_{i}}{dt} & = & D_{2}\Delta R_{i}+b-eR_{i}-R_{i}\cdot \left(k_{1}+k_{2}R_{a}^{2}A\right)+cR_{a} \end{array}$$

where *D*_1_ and *D*_2_ are the diffusion rates of active and inactive ROP, respectively. *a* is the production rate of active ROP, *b* the production rate of inactive ROP while *r* is the degradation rate of active ROP and *e* is the degradation rate of inactive ROP. Further, we have also included a degradation of active ROPs at the cell boundary (compartments that have the background as a neighbor) with a rate *p*, corresponding to active ROPs diffusing to the anticlinal sides or out of the cell. We assume that ROP is only produced in its inactive form and only degraded in its active form (Table 1). Transport between compartments is assumed to be proportional to the difference in concentrations, with spatial factors being included in the diffusion constant. The auxin simulation was run first, and the resulting auxin gradient was used in the ROP simulation. Both simulations were run until the system was in equilibrium. We discretized the 2D surface into 286 polygonal compartments, and spatial factors are added to the transport rates given the different sizes of the compartments and their neighbor cross sections. All simulations use a 4th order Runge-Kutta solver and were implemented in an in-house developed open source software (http://dev.thep.lu.se/organism), available upon request. Files defining the models, the initial configuration, and the solver parameters are provided as Supplementary Information.

**Table 1:** **Model parameters for the ROP activation model**.

**Symbol**	**Value**	**Description**	**Figures**
*D*_*a*_	5.0 length^2^/s	Diffusion rate of auxin	Figure 2A
*t*	0 conc/s	Production of auxin throughout the cell	Figure 2A
*t*	0.11 conc/s	Production of auxin throughout the cell	Figure 2B
*t*	0.12 conc/s	Production of auxin throughout the cell	Figure 2C
*t*	0.13 conc/s	Production of auxin throughout the cell	Figure 2D
*s_in_*	0.25 conc/s	Auxin source production rate	Figure 2A
*s_out_*	0.31/s	Auxin sink degradation rate	Figure 2A
*q*	2.0·10^−5^ 1/s	Degradation rate of auxin	Figure 2A
*q*	0.1 1/s	Degradation rate of auxin	Figures 2B–D
*D*_1_	0.01 length^2^/s	Diffusion rate of active ROP	Figures 2A–D
*a*	0 conc/s	Production rate of active ROP	Figures 2A–D
*r*	0.01 1/s	Degradation rate of active ROP	Figures 2A–D
*p*	0.01 1/s	Rate of boundary degradation of active ROP	Figures 2A–D
*k*_1_	0.01 1/s	Rate of constant ROP activation	Figures 2A–D
*k*_2_	0.015 1/(conc^3^ s)	Rate of auxin-dependent ROP-autoactivation	Figures 2A–D
*c*	0.1 1/s	Rate of constant ROP inactivation	Figures 2A–D
*D*_2_	1.0 length^2^/s	Diffusion rate of inactive ROP	Figures 2A–D
*b*	0.01 conc/s	Production rate of inactive ROP	Figures 2A–D
*e*	0 1/s	Degradation rate of inactive ROP	Figures 2A–D

**Figure 2:**
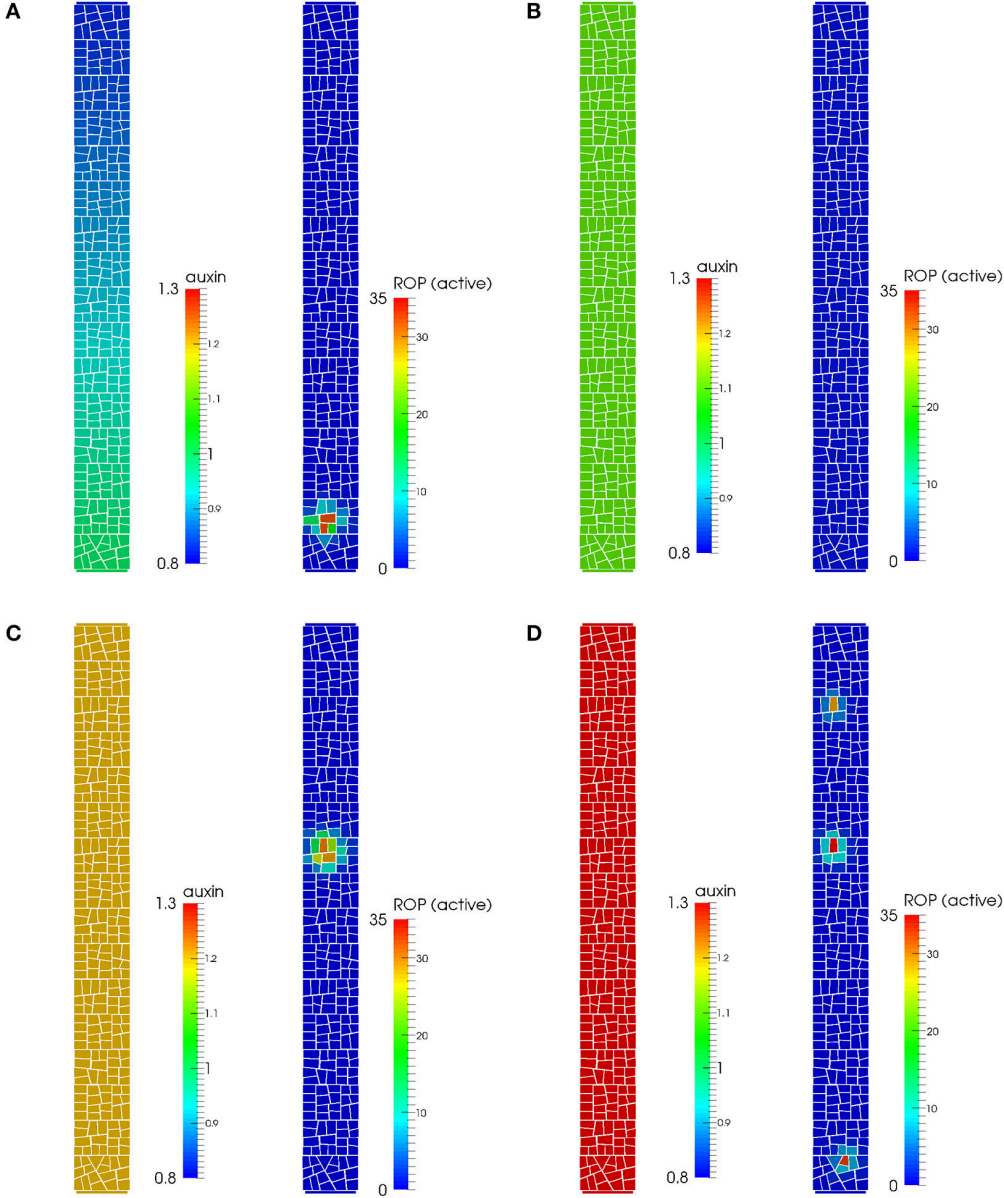
**Auxin gradient and pattern of active ROP in a model of auxin-driven ROP-activation**. **(A)** When an auxin gradient is present, active ROPs can localize centrally toward the basal end along the outer membrane of the epidermal cell, similar to the experimentally observed pattern. **(B)** A low activation of ROPs is seen for a constant auxin level of 1.1, with no clear peaks of active ROP forming. **(C)** For a constant auxin level of 1.2, the active ROP peak localizes closer to a middle position along the cells apical-basal axis. **(D)** For a constant auxin level of 1.3, several ROP peaks appear throughout the cell.

*For the simulations with constant auxin gradient in Figures 2B–D, the parameters D_a_, s_in_, s_out_ are all set to zero*.

### 2.3. Mechanical simulations and material model

The model treats the epidermal wall of a root cell as a thin shell under turgor pressure. We want to focus on the site of root hair outgrowth and analyze the trends in the stress pattern around the root hair outgrowth site under different hypotheses. Thus, the model consists of a square patch of dimensions 20 × 20μ*m* and thickness of 0.5μ*m*. The boundaries of the patch were fixed in place and the patch was under pressure load perpendicular to its surface at all times. We used finite element models for all mechanical simulations using linear quadrilateral (S4R) and triangular (S3) shell elements in Abaqus (Dassault Systemes, 2012). We employed general static analysis with adaptive stabilization and included nonlinear geometric effects.

The finite element method is based on linearization of the virtual work, δ*W*, equation

(4) $$\begin{array}{l} \delta W=\int_{V}S:\delta \dot{E}dV-\int_{V}f_{0}\cdot \delta vdV-\int_{\partial V}t_{0}\cdot \delta vdA=0, \end{array}$$

where *S* is a second Piola-Kirchhoff stress tensor and Ė is the rate of change of its work conjugate Green-Lagrange strain tensor. The two last terms of Equation (7) contribute to the external virtual work component and *f*_0_ and *t*_0_ represent body force per undeformed unit volume and traction per undeformed unit area, respectively. For hyperelastic materials second Piola-Kirchhoff stress tensor can be calculated from strain energy function *U* as a derivative with respect to Green-Lagrange strain tensor

(5) $$\begin{array}{l} S=\frac{\partial U}{\partial E}. \end{array}$$

In Saint Venant-Kirchhoff model strain energy function takes the form

(6) $$\begin{array}{l} U=\frac{1}{2}\lambda {\left(trE\right)}^{2}+\mu E:E, \end{array}$$

where λ and μ are Lamé coefficients related to Young's modulus *E*_*Y*_ and Poisson ratio ν by formulas

(7) $$\begin{array}{rcl} E_{Y} & = & \frac{\mu }{\lambda +\mu }\left(2\mu +3\lambda \right), \end{array}$$

(8) $$\begin{array}{rcl} \nu & = & \frac{\lambda }{2\left(\lambda +\mu \right)}. \end{array}$$

We applied standard isotropic elastic material in Abaqus with elastic modulus of 100 MPa. In softened regions we used Young's modulus of 70 MPa and in stiffened regions 130 MPa. We assumed turgor pressure of 0.2 MPa and increased turgor pressure in central region of 0.4 MPa. In all cases we used Poisson ratio of 0.2. The experimental estimates of plant cell wall elasticity from *in vivo* samples (Suslov et al., 2009; Hayot et al., 2012; Nezhad et al., 2013) and synthetic bio-composites (Chanliaud et al., 2002) cover the large range 100 kPa to 1 GPa depending on the type of plant tissue and measurement method. Similarly the turgor pressure measurements can vary from 2 to 10 atm. For simulations we have chosen elastic modulus in the middle of this range. The turgor pressure was chosen such that the wall deformation is macroscopic but not exaggerated and turned out to be on a lower side of the experimental range. We took into account the influence of the atmospheric pressure so the load pressure in the simulation is a turgor lowered by 1 atm.

The Saint Venant-Kirchhoff material model is in principle a simplistic model of a real plant wall material in the sense that it does not reflect its complicated nonlinear visco-elastic and plastic properties. However, in this case we are interested rather in general stress pattern changes than in the accurate description of deformations and thus the linear material model presents a simple alternative with a well understood notion of elastic modulus.

## 3. Results

The results reported in this communication present two connected mechanisms concerning root hair outgrowth. Firstly we consider the process by which the site of the root hair outgrowth can be specified within a cell, by the localization of activated ROP into a small patch. Secondly we examine if the initiation of root hair growth can be explained by mechanical perturbations in such a patch. We consider several scenarios and analyze the emerging pattern of stresses in comparison with experimentally observed microtubule organization.

### 3.1. An auxin-driven ROP-activation model can guide the activated membrane-localized ROP into a basally localized patch in the 2D epidermal outer cell membrane

We developed a 2D single cell model where the cycling of ROP from an inactive to an active form is influenced by an auxin gradient (Methods, Red box in Figure 1A). The model is an extension of a previously published 1D model of ROP cycling (Payne and Grierson, 2009). We discretize the cell into several compartments between which the ROPs are allowed to diffuse, assuming a faster movement of inactive ROPs, which reside in the cytosol, compared to the active ROPs which are connected to the membrane. In contrast to the model which it is based on, we have explicitly modeled the auxin gradient as resulting from diffusion of the auxin molecule with a source of auxin production at the basal end of the cell and an auxin sink, where auxin is degraded, at the apical end. We assume that ROPs are created in their inactive form and subsequently activated by auxin to become the active membrane-bound form. Further, the active form of ROP is subject to constant degradation. Also included in the model is a non-linear self-activation of ROP. All reactions follow simple mass action and diffusion descriptions (Equations 1–5).

First we tested whether such a molecular model is able to create a peak of active ROP, marking the site of root hair outgrowth, at the correct location in the epidermal cell membrane. We expect the peak to locate close to the basal end of the lateral membrane, even when considering a full 2D description of this membrane. Indeed, a patch of active ROP localizes at the basal end of the lateral membrane (Figure 2A), slightly away from the cell wall, consistent with previous experimental findings (Molendijk et al., 2001; Jones et al., 2002; Fischer et al., 2006). The patch first appears near the cell boundary where the level of auxin is predicted to be highest, after which it moves a small distance away from the cell boundary where it becomes stable. To confirm the importance of the auxin gradient for the localization of the peak, we simulated the model with constant auxin in the cell (Figures 2B–D). The basal bias for the ROP patch is lost, and depending on the auxin level, a single central peak, several peaks spread across the cell, or a low activation of ROP throughout the cell was found. Interestingly, phenotypes as multiple hairs, more apical root hair positions and loss of root hairs have been found in mutants suggested to alter intracellular auxin levels and or gradients (Masucci and Schiefelbein, 1994; Grebe et al., 2002; Fischer et al., 2006; Ikeda et al., 2009). The model parameters of the simulation with a gradient were set such that they generate a gradient of about 20%, showing that the gradient does not need to be steep to generate enough bias for the ROP dynamics. While the intracellular gradient has yet to be measured in experiments, the gradient is well within ranges suggested in tissue models of auxin in the root (Swarup et al., 2005; Jones et al., 2009).

Our model confirms, in a 2D setting, that a sub-cellular auxin-dependent activation of ROPs promoted by an intracellular auxin gradient together with intracellular transport is sufficient to create convergence of active ROPs, placing the site of root hair formation to the center close to the basal end of the outer epidermal cell plasma membrane. The active ROP is an early marker of root hair initiation and we will use this to investigate how such a patch may influence mechanical properties of the cell wall such that a root hair can be initiated, and whether this can lead to a star-like pattern of stresses.

### 3.2. Altering mechanical stiffness locally at the root hair initiation site can guide stresses from circumferential to radial

The mechanical aspects of root hair growth are analyzed by means of a finite element model of the epidermal wall of a rectangular cell and from changing material properties in a small region representing an activated ROP patch.

**Figure 3:**
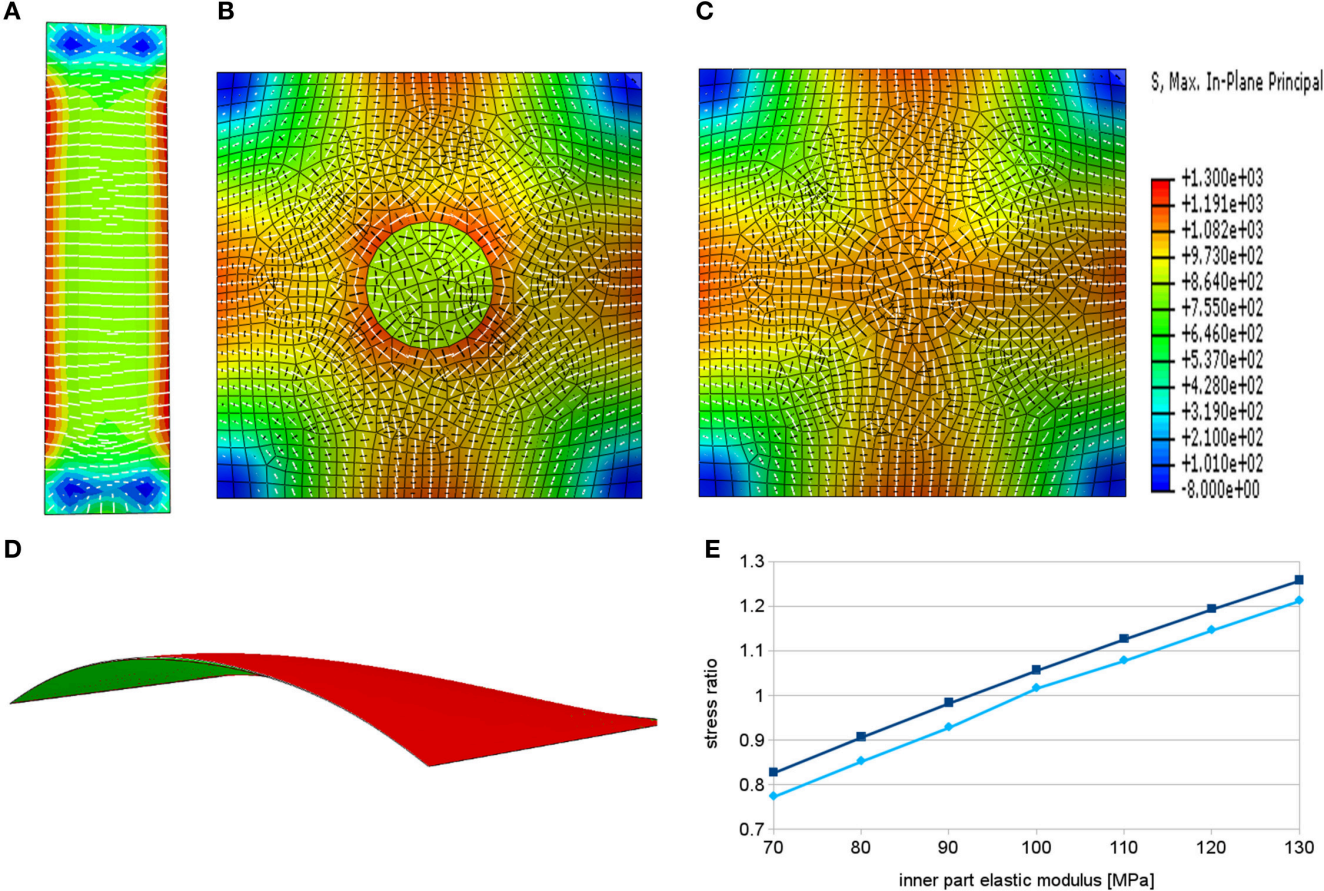
**Principal stress directions predicted by finite element models in root hair cells**. White bars show the direction of the maximal principal stress, the black bars indicate minimal principal stress directions and the color represents the maximal stress magnitude. **(A)** Pattern of maximal principal stress in the epidermal wall of a pressurized rectangular cell bears resemblance to microtubular patterns of an approximately rectangular root epidermal cell except in the region of subsequent root hair outgrowth. **(B)** In case of softened material in the center of the patch we observe circumferential alignment of maximal principal stress around this region. **(C)** Increasing the elastic modulus of the material in the same region leads to radial organization of maximal principal stress around the center. **(D)** Cross section through the pressurized model showing the curvature of the surface and a slight difference in deformation between the simulation with the softened center (red) and the hardened center (green). **(E)** Graph showing dependence of the ratio of radial to circumferential stress components on the elastic modulus of this part in two points around the region of modified material in the center. The elastic modulus of the remaining part of the model was set to 100 MPa. The ratios below one correspond to the dominant circumferential stress direction while the ratios above one signify the dominant radial stress direction.

A simulation of rectangular epidermal wall under turgor pressure results in a stress pattern in which the first principal stress component is mostly oriented perpendicularly to the long axis of the cell (Figure 3A). This pattern correlates well with the orientation of microtubules observed in close to rectangular epidermal cells of the *Arabidopsis* root (Figure 1B, Pietra et al., 2013). Note that this result pertains to the cell scale stresses and is independent of the root tissue curvature where a pressurized cylindrical root shape would also produce highest stresses in the circumferential direction, e.g., Bozorg et al. (2014). Hence, the simulation suggests that cellular stresses can complement tissue scale stresses to provide a directional cue for microtubules in roots and other elongated tissues with elongated cells. Note that there are deviations in the general stress patterns in the proximity of basal and apical ends of the outer wall of the root hair cell (Figure 3A). Such a pattern could provide a mechanical bias for root hair initiation, but the effect can be dependent on the specific choice of material model.

More intriguingly, in experiments deviation from this pattern of microtubule orientation appears at the site of subsequent root hair outgrowth, where a star-like pattern around the initiation point can be observed (Figures 1C,D, Pietra et al., 2013). We extended the model to analyze whether mechanical perturbations in a localized patch can reconcile the experimental observations of microtubule organization with stress patterns surrounding the patch. We localize the site of the root hair outgrowth to a circular region, which can have different mechanical properties. We assume that the outer edges of the cell are fixed in space and the loading forces arise from turgor pressure. We have previously shown that an assumption of local loosening of the pressurized cell wall(s) leads to a circumferential pattern of tissue scale stresses surrounding the loosened region (Hamant et al., 2008). A similar principle applies for the simulation of local loosening of a root epidermal cell wall (Figure 3B), which shows circumferential maximal principal stress around the loosened region. Such loosening is suggested to be a prerequisite, for example, for the diffuse growth in plant meristems and it is supposed to be a result of breaking the bonds that link the cellulose fibers or of processes that affect the pectin matrix (Cosgrove, 2005; Braybrook and Jönsson, 2016).

In tip growing cells rapid deposition of new wall material and complex pectin chemistry may alter mechanical properties at the tip (Bosch, 2005; Pang et al., 2010; Rounds and Bezanilla, 2013). We hypothesize that such rapid deposition of new material and reorganization of cell wall components can, at least temporarily, lead to local stiffening of the cell wall. Indeed, under the assumption of local stiffening of the material we obtain a radial pattern of maximal stress in the surrounding region (Figures 3C,E), which is matching the microtubule pattern seen *in vivo* (Figure 1C, Pietra et al., 2013). This simulation suggests a phase of local stiffening, by addition of more wall material or by changes to the wall properties, preceding the localized growth phase of the root hair. Interestingly, such suggestion is in parallel with observations of changes in thickness of the cell wall in pollen tube tips, which show oscillatory behavior and thickening prior to the growth phase (McKenna et al., 2009). It is worth to point out that the opposing hypotheses about the change of material stiffness at the outgrowth site would produce different types of elastic deformation. In case of softening of the material, we would expect bulging out of the surface of the tip and in case of hardening of the material, we should observe flattening of the surface. This effect could be small and transient, as it can be overshadowed by the rapid growth process, and thus hard to observe experimentally, but potentially it could be used to discern between the two cases (Figure 3D).

We explored the idea that the quick addition of material connected to root hair initiation might, at least transiently, lead to stiffer walls at the initiation site, and our model predicted radial stresses surrounding such a region. In such scenario stresses correlate with the star-like microtubule patterns seen in root hair cells before root hairs grow out.

### 3.3. Heterogeneous forces can generate radial stress patterns surrounding a root hair initiation site

**Figure 4:**
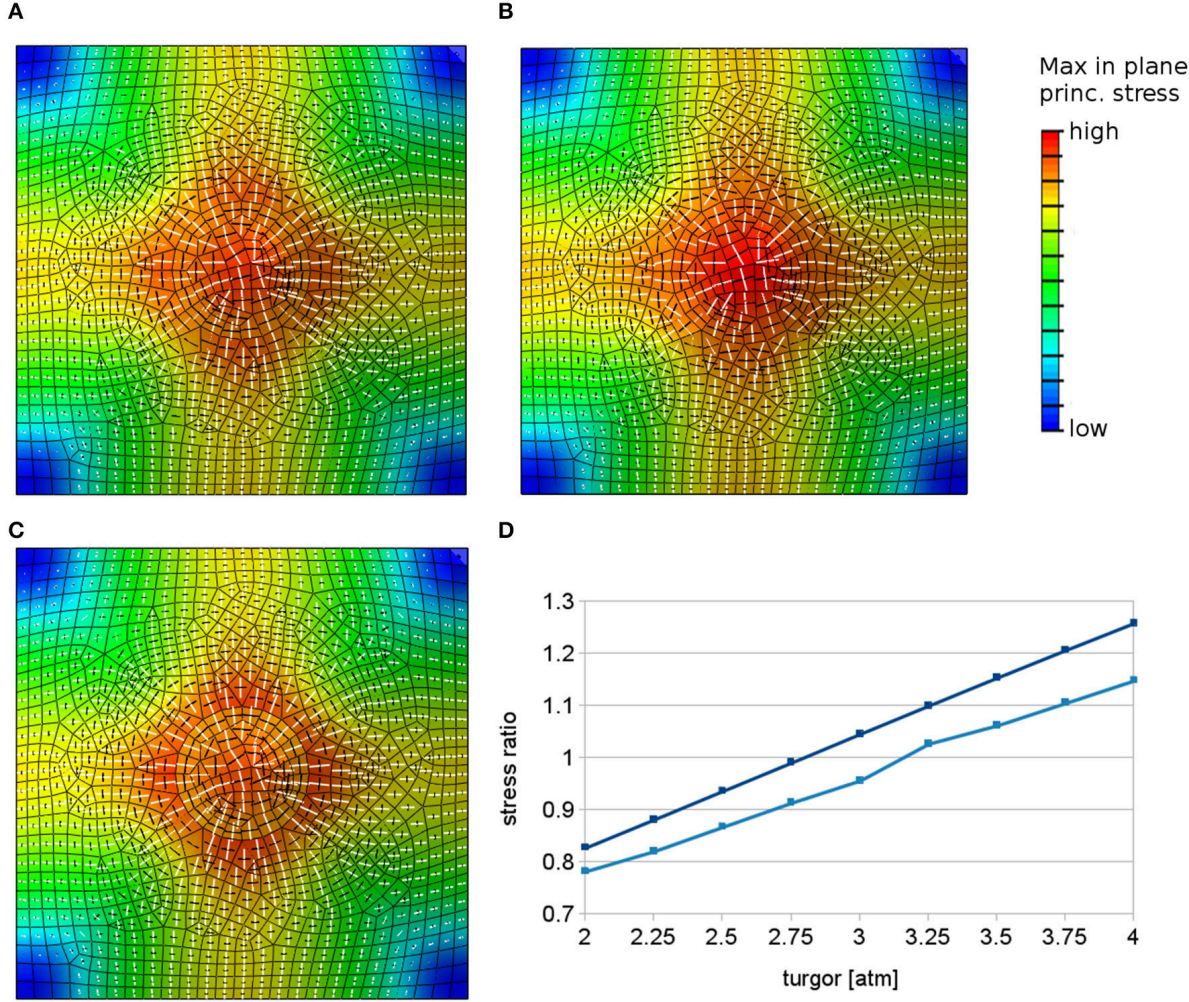
**Simulations with heterogeneous loading forces**. White and black bars show respectively maximal and minimal principal stress directions in finite element models when loading forces are locally increased in the region of predicted root hair outgrowth. The color represents the maximal stress magnitude. **(A)** Principal stress directions predicted by finite element models in the case of locally increased pressure in the center when material properties are kept constant. **(B)** The radial pattern can appear also when the material in the patch is made elastically softer. **(C)** The mechanisms yielding radial stress alignment can be combined without destruction of the radial stress pattern. The image presents a combination of elastically stronger center with locally increased forces. The radial pattern of maximal principal stress is still evident. **(D)** Graph showing the trend of the stress anisotropy vs. the inner region pressure value measured at two different points on the boundary of the circular region, where the pressure is increased and the material is made softer with respect to the rest of the surface. The principal stresses change from circumferential (values below 1) to radial (values above 1).

Another mechanism that may contribute to tip growth is a differential pressure model (Winship et al., 2010; Zonia, 2010), possibly driven by strong cytosolic streaming together with heterogeneous cytoskeletal crowding. While a pressure difference within a root epidermal cell might be hard to envision, a heterogeneous force distribution at the wall might still be possible, where for example the cytoskeleton could exert forces on the site of outgrowth leading to increased loading of this region. Application of increased outward forces in a patch can lead to radial stresses around the outgrowth site in simulations (Figure 4A). We increase loading forces by increasing pressure in the small region in the simulations up to 200% of the pressure value in the remaining part of the cell. This large pressure difference that is required to change the main principal stress pattern to radial around the outgrowth site (Figure 4D) might be hard to justify biologically by just cytosolic streaming and makes this hypothesis questionable.

Next we test a combination of the previously analyzed mechanisms of local material or loading force changes during root hair initiation. Strikingly, locally increased forces at the site of outgrowth can lead to radial stress pattern even in the case of elastically softened material in the outgrowth region (Figure 4B, cf. Figure 3B). This however depends on the relation between the difference in Young's modulus and pressure in both regions in such a way that there exists a threshold where radial stress pattern occurs. This possibility of combining local material softening with locally increased forces at a tip growth site allows for a mechanism in which the structure of the cell wall changes, allowing greater wall extensibility similarly to the scenario suggested for diffuse wall growth and, at the same time, local forces exerted by the cytoskeleton contribute to tip growth.

Finally, if locally increased forces and local material stiffening are combined, a slightly stronger (more anisotropic) radial stress pattern results (Figure 4C). This scenario can be of interest since there is the possibility that cytoskeleton reorganization during tip growth itself leads to local stiffening of the cell wall material.

In summary, the finite element model predicts that radial stress patterns are possible surrounding a small region where increased forces are applied. This can be realized independently of any heterogeneous or anisotropic material properties in such a region.

## 4. Discussion

The importance of growth for morphogenesis in plants has led to a large interest in how cortical microtubules organize into patterns regulating cellulose deposition and subsequent growth. The classic model is that the microtubules organize like hoops around a barrel to generate anisotropic growth (Green, 1962).

Our study was inspired by the strikingly different pattern of microtubules seen at the initiation of root hairs, where a radial pattern is found around the initiation site (Figure 1, Pietra et al., 2013). Importantly, we acknowledge that the root hair initiation process involves a complex combination of molecular and mechanical patterning (Figure 1A). Hence, our first aim was to investigate a mechanism for marking the site of the root hair outgrowth in a molecular 2D model based on a previous 1D effort (Payne and Grierson, 2009). An early marker for the site where a root hair is initiated is a peak of active ROP protein (Jones et al., 2002). Our simulations demonstrate that an internal auxin gradient promoting ROP activation together with self-activating feedback is sufficient to correctly place the peak centrally at the basal side of the lateral membrane (Figure 2A).

We then investigated how a localized change in mechanical properties affects stresses surrounding this region, in particular if a radial star-like pattern of microtubules (Figure 1C, Pietra et al., 2013) can be predicted by stress patterns. This appeared plausible since it has previously been reported that microtubular patterns correlate with maximal stress directions at subcellular and at tissue scales (Hamant et al., 2008; Sampathkumar et al., 2014). For example, the outgrowth of primordia at the shoot apical meristem leads to a circumferential pattern of microtubules and the intracellular patterns of stresses can be used to predict microtubular patterns in the complex shapes of leaf epidermal pavement cells (Sampathkumar et al., 2014). Of course this correlation does not mean that the microtubules organize according to stress patterns in all cases and systems. It is possible and likely that other mechanisms not involving mechanical inputs are involved in microtubule organization. Here we analyze which mechanical conditions have to be realized to explain the available microtubule data on the basis of a microtubule-stress alignment hypothesis.

We presented two different scenarios that could lead to a radial pattern of stresses during tip growth, reconciling the alignment of microtubules and stresses in the case of root hair initiation. Firstly, the quick addition of material could lead to a stiffening of the wall, and we could show that this can lead to radial stress patterns (Figure 3C). This can be related to observations of alternating phases of tip growth and wall thickening at the tip (McKenna et al., 2009). Although this may occur at a different time scale during root hair initiation, only about 50% of analyzed cells showed a radial pattern, which could indicate that it represents a transient state (Pietra et al., 2013). A competing idea suggested for tip growth is that the forces exerted on the wall at the tip are changing (Zonia, 2010). When applied to a model of a patch in the epidermal wall, this was also able to generate radial patterns of stresses (Figure 4), but the required difference in forces was high and might be hard to realize in reality in an epidermal root cell (Figure 4D, Winship et al., 2010).

Since our results show that either local alteration of material properties of the cell wall or the active interaction with cytoskeleton may lead to the radial pattern of stresses around the place of root hair outgrowth, it would be interesting to measure wall stiffness at this site for example by using atomic force microscopy.

While we have stressed the importance of looking at several processes when analyzing root hair initiation (Figure 1A), our computational simulations have been divided into the processes of ROP patch formation (Figure 2) and of ongoing mechanical changes (Figures 3, 4). A main challenge will be to integrate these into a single model where both ROP activation is necessary for root hair initiation (Jones et al., 2002), and correct microtubular dynamics are necessary for correct ROP positioning (Pietra et al., 2013). Induced chemical or genetic perturbations followed by live imaging can provide additional dynamical data to generate improved insight into the process, and computational modeling of the interactions will be essential to understand the consequences of direct or indirect mechanisms of several combined feedback regulations.

## Author contributions

SP and MG designed and analyzed experiments. PK, AL, BB, HJ designed and analyzed models and simulations. PK and BB developed and simulated mechanical models. AL developed and simulated the molecular model. All authors wrote and edited the paper.

## Funding

This work was funded by the Knut and Alice Wallenberg Foundation via grant ShapeSystems (KAW 2012.0050) to MG and HJ, the Swedish Research Council (VR2013-4632) to HJ, and the Gatsby Charitable Foundation (GAT3395/PR4) to HJ.

### Conflict of interest statement

The authors declare that the research was conducted in the absence of any commercial or financial relationships that could be construed as a potential conflict of interest.
